# Kidney volume, kidney function, and ambulatory blood pressure in children born extremely preterm with and without nephrocalcinosis

**DOI:** 10.1007/s00467-019-04293-9

**Published:** 2019-07-23

**Authors:** Alexander Rakow, Åsa Laestadius, Ulrika Liliemark, Magnus Backheden, Lena Legnevall, Sylvie Kaiser, Mireille Vanpée

**Affiliations:** 1 Neonatal Unit, Department of Women’s and Children’s Health, Karolinska Institutet, Karolinska University Hospital, 17176 Stockholm, Sweden; 2 Pediatric Nephrology Unit, Department of Women’s and Children’s Health, Karolinska Institutet, Karolinska University Hospital, Stockholm, Sweden; 3 Unit for Medical Statistics, Karolinska Institutet, Stockholm, Sweden; 4 Pediatric Radiology Unit, Department of Women’s and Children’s Health, Karolinska Institutet, Karolinska University Hospital, Stockholm, Sweden

**Keywords:** Blood pressure, Circadian regulation, Kidney volume, Nephrocalcinosis, Preterm, Renal function

## Abstract

**Background:**

Reduced kidney volume (KV) following prematurity is a proxy for reduced nephron number and is associated with the development of hypertension and end-stage renal disease in adults. We investigated whether extreme prematurity affects KV, function, and blood pressure in school-aged children and if nephrocalcinosis (NC) developed during the neonatal period had additional effects.

**Methods:**

We investigated 60 children at a mean age of 7.7 years: 20 born extremely preterm (EPT < 28 weeks gestational age with NC (NC+)), 20 born EPT without NC (NC−), and 19 born as full-term infants (control). We measured KV by ultrasound, collected blood and urine samples to evaluate renal function, and measured office and 24-h ambulatory blood pressure (ABPM).

**Results:**

Children born EPT had significantly smaller kidneys (EPT (NC+ NC−) vs control (estimated difference, 11.8 (CI − 21.51 to − 2.09 ml), *p* = 0.018) and lower but normal cystatin C–based glomerular filtration rate compared with control (estimated difference, − 10.11 (CI − 0.69 to − 19.5), *p* = 0.035). KV and function were not different between NC+ and NC− groups. Change in KV in relation to BSA (KV/BSA) from the neonatal period to school age showed significantly more EPT children with neonatal NC having a negative evolution of KV (*p* = 0.01). Blood pressure was normal and not different between the 3 groups. Fifty percent of EPT had a less than 10% day-to-night decline in ABPM.

**Conclusions:**

Kidney growth and volume is affected by EPT birth with NC being a potential aggravating factor. Circadian blood pressure regulation seems abnormal in EPT-born children.

## Introduction

Extreme prematurity interferes with nephrogenesis, leaving each individual with a finite number of nephrons which might increase the risk for reduced functional capacity and the later development of impaired renal function and hypertension [1–3]. Since Brenner et al. introduced the concept of low nephron number at birth leading to further loss of nephrons by hyperfiltration and glomerulosclerosis, multiple studies have described the association between low birth weight and kidney size, kidney function and blood pressure, mostly in adolescents and adults but also in children [1, 4–6]. There is insufficient data to conclude whether prematurity itself is the single adverse event or if additional factors during the neonatal period, such as extra-uterine growth restriction, nephrocalcinosis (NC), nephrotoxic drugs, sepsis, and hypotension, have additional effects on kidney function and the risk for later renal failure, renal hypertension, and overall cardiovascular morbidity and mortality.

NC is defined as the pathological deposition of calcium crystals in the renal parenchyma. The incidence of neonatal NC varies between 7 and 64% depending on ultrasonographic criteria and is highest in the most premature infants [7–9]. Ultrasound has been found to be a sensitive and reliable method to detect NC [10]. The etiology of NC in preterm neonates has not been fully clarified. Furosemide has frequently been implicated as a causative factor due to its hypercalciuric effect [11]. Aminoglycosides, corticosteroids, and xanthenes have also been identified as potential risk factors for NC [12]. Besides nephrotoxic drugs, the preterm infants often experience severe infections, hypotensive crisis, and hypoxia as well as hemodynamic impairment by a persistent ductus arteriosus and/or its treatment, all of which potentially lead to transient or even permanent renal failure [13].

Nutrition is suspected to have an important impact on early postnatal kidney development and health. Recent research suggests that high protein intake, advocated for better growth velocity for extremely preterm (EPT) infants, might have a disadvantageous effect on the kidney. It has been suspected that this high-risk group could have difficulties in metabolizing the amount of protein given, leading to mild metabolic acidosis and possibly to hypercalciuria [14]. Also, the improvements in support of micronutrients to enteral and parenteral feeds including additional calcium, phosphate, and vitamin D bear a potential risk for an imbalance towards stone-promoting factors [15].

The current evidence indicates that neonatal-acquired NC resolves by 50% during the first year of life and to 75% by school age without having an impact on kidney function [11, 16]. However, from the few studies focusing on this subject, it can be suspected that NC has a detrimental effect on the kidneys and therefore cardiovascular health later in life [9, 17].

In this study, we investigate whether NC developed during the neonatal period in children born EPT has an impact on kidney volume and function at school age. Eventual effects on blood pressure are evaluated by 24-h blood pressure monitoring (ABPM).

## Subjects and methods

### Subjects

The study was approved 5by the Ethical Committee at Karolinska University Hospital. Written and oral consent was obtained from all parents and children.

**Fig. 1:**
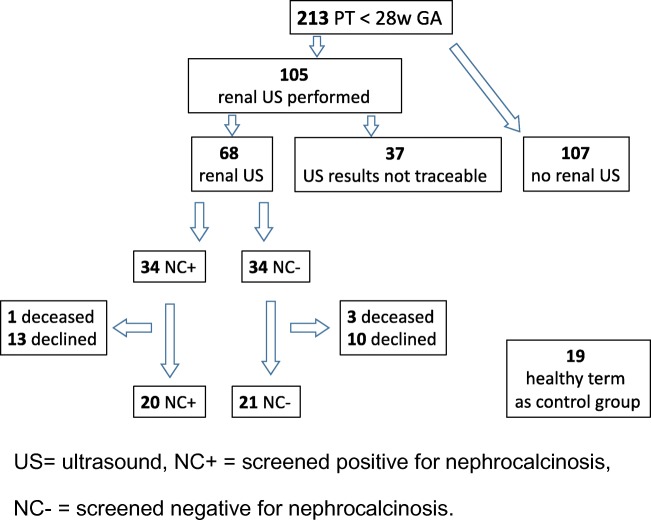
Flowchart for patient inclusion for extremely preterm infants born < 28 weeks (w) gestational age (GA) from 2008 to 2011 at Karolinska University Hospital, Stockholm, Sweden, in numbers. US, ultrasound; NC+, screened positive for nephrocalcinosis; NC−, screened negative for nephrocalcinosis

We identified 213 infants born before 28 weeks gestational age (GA) between 2008 and 2011 at the Karolinska University Hospital, Stockholm, Sweden (Fig. 1). Neonatal renal ultrasound was performed in 105 infants, but only 68 had traceable results and images. All neonatal investigations were performed by pediatric radiologists. NC was defined as hyper-echogenic reflections in cortex and or medulla visualized in longitudinal and transverse projections. Of the investigated 68 infants, 34 were diagnosed with NC (NC+) during their late neonatal period and 34 infants showed no signs of NC (NC−). There was no history of hyperoxaluria, cystinuria, or any type of renal tubular acidosis or a history of antenatal or postnatal diagnosis for urogenital malformation in any patient. Twenty-three families refused to participate and 4 children died after discharge from the neonatal unit. Twenty children with NC and 21 without NC during their neonatal period consented to participate in the study. The 172 non-participants (children without ultrasound investigation and children with ultrasound investigation but lost to follow-up or declined to participate or those with incomplete images for review) were not different from the participants with regard to perinatal characteristics. A total of 19 healthy children born at term with appropriate birthweight, without any congenital abnormalities, and with no history of kidney diseases selected from delivery room records were recruited as controls. All children were in good health at the time of the visits.

### Follow-up visit

Clinical data was collected from the neonatal charts with particular attention to factors that could influence renal function, such as nephrotoxic substances: aminoglycosides, vancomycin, loop diuretics, thiazide diuretics, and antenatal and postnatal steroids. GA, birth weight, Apgar scores, intrauterine growth retardation, respiratory distress syndrome (RDS), bronchopulmonary dysplasia (BPD) as defined by NIH, acute kidney injury (AKI) defined and staged by the KDIGO guidelines, patent ductus arteriosus (PDA) requiring treatment, sepsis episodes (clinical and/or culture verified), necrotizing enterocolitis (NEC) Bell stage II or more, surgical interventions for NEC, retinopathy of the premature (ROP) grade III or higher (and or any plus disease), and intraventricular hemorrhage (IVH) or parenchymal hemorrhage as defined by Papile were documented. Small for gestational age (SGA) was defined as a birth weight < − 2 standard deviations (SD) according to Swedish reference data for normal fetal growth [18].

At the visit, patient and parental medical histories, as well as maternal and paternal height and weight, were registered. The same research nurse performed all anthropometric measurements for weight, height, head circumference, and waist circumference on the children, who were wearing light indoor clothing. Height was measured using a wall-mounted stadiometer. Waist circumference was measured midway between the lower rib margin and the iliac crest using a normal measuring tape. Body mass index (kg/m^2^), body surface area (BSA = 0.007184 × Height^0.725^ × Weight^0.425^) [19] and waist-to-height ratio were calculated.

Office blood pressure was measured in all patients using an automated oscillometric device (GE Healthcare Dinamap Carescape V100). After a 30-min rest, three consecutive measurements were taken on the child’s non-dominant arm with an appropriate cuff size (bladder width that was at least 40% of the arm circumference [20]). We used a SPACELABS 90217A (SpaceLabs Medical Inc., Redmond, Washington, USA) device for ABPM, using the same cuff size as for the office measurements. The device was programmed to register blood pressure every 20 min between 07:00 AM and 21:00 PM and every 60 min during the night (21:00 PM–07:00 AM). Due to compliance difficulties in 21 children (12/20 NC+, 9/20 NC−) with neuropsychiatric disorder (ADHD, autism, and autism spectrum), we accepted > 50% of successful readings instead of the standard > 75% for statistical analysis [21]. The rest periods were described by the patient’s parents, and day and night measurements were adjusted accordingly. The non-dipper pattern was defined as nocturnal BP_Systolic_ or BP_Diastolic_ < 10% relative to the diurnal mean value [22]. Hypertension was defined as > 95th percentile according to gender and length. BP > 90th but < 95th percentile was defined as high normal BP [22].

### Renal function estimation

Morning urine samples were collected from all study participants and analyzed for sodium, potassium, creatinine, albumin, calcium, phosphate, magnesium and protein HC (α-1-microglobulin) and immunoglobulin G. Sodium and potassium were measured using potentiometry with ion-selective electrodes (Cobas 8000, Cobas C ISE2, Roche, Basel Switzerland). Urine phosphate, calcium, and magnesium were measured using photometric technique (Cobas 8000 Cobas CC 701 Roche, Basel Switzerland), and urine albumin was measured using immunochemical and turbodimetric method (Cobas 8000 Cobas CC 701 Roche, Basel Switzerland). Protein HC and immunoglobulin G were measured by immunochemical and nephelometric method (BN Pro Spec, Siemens Healthcare, Erlangen, Germany).

Blood samples were taken in 55 patients (NC+,17; NC−, 21; control, 17) after placing a local application of a topical anesthetic cream containing 2.5% lidocaine and 2.5% prilocaine (EMLA; Astra Zeneca, Sodertalje, Sweden). Blood samples were investigated for plasma sodium and potassium using potentiometry with ion-selective electrodes (Cobas 8000, Cobas C ISE2, Roche, Basel Switzerland); calcium, phosphate, alkaline phosphatase, creatinine, and urea using photometric technique; and cystatin C using immunochemical and turbidometric method (Cobas 8000 Cobas CC 701 Roche, Basel Switzerland). For the estimation of GFR, we used the simplified Schwartz formula (with *k* = 36) as well as the cystatin C–based CAPA formula [23, 24].

### Assessment of renal volume

Ultrasound of the kidneys during the neonatal period (2008–2011) was performed by pediatric radiologists using a Siemens S 2000 with a 6C2 curved transducer (Siemens, Erlangen, Germany) at an average age of 36 weeks postmenstrual age following local guidelines. All images from the neonatal period for the 41 included children were reviewed by a senior pediatric radiologist in 2018. Diagnosis of NC was confirmed in 39 of the 41 cases. In each group (NC+/NC−), one patient was misdiagnosed and moved to the opposite group. Kidney volume for the neonatal period was calculated in 36 out of the 41 patients using the equation for an ellipsoid described elsewhere [25] and expressed as a ratio to BSA (KV/BSA) [26]. In the remaining 5 patients, the reviewed images were incomplete and volume calculation therefore was not possible.

Ultrasound of the kidneys at school age visits was performed by a single experienced user. All investigations were performed with a Philips EPIQ 7G with SW1.5.2 software (Philips Ultrasound, Inc. 22100 Bothell Everett Hwy Bothell, WA 98021-8431, USA) using a C9-2 curved transducer. Multiple (at least two) measurements of kidney length, width, and depth were performed. An average of these measurements was entered in the equation for an ellipsoid, and KV was calculated [25]. Results from volumetric kidney measurements were adjusted for BSA by using linear regression analysis but also by using the ratio of kidney volume (KV) to BSA [26]. Predicted KV was calculated for each individual using the equation described by Dinkel et al. [25].

### Statistics

For descriptive statistics, continuous variables were presented with mean and standard deviation (SD). Continuous variables, approximately normally distributed, were analyzed with respect to the three defined groups, using analysis of variance (ANOVA). In order to adjust for continuous prognostic variables, covariance analysis was used (ANCOVA). Stepwise regression analysis was used to examine the impact of a set of prognostic variables. The coefficient of determination, R^2^ was used to compare the precision of different models. Non-normal continuous variables were analyzed with Kruskal-Wallis test. Dichotomous variables were analyzed with cross tables and Pearson’s chi-square test. As additional analyses, the kidney volume was analyzed with a mixed effects model including right and left volume in the same analysis. A hierarchical model with the child as the main unit was set up, taking into consideration the correlation between right and left side, with the covariance structure unstructured (UN). In all statistical analyses, the relevant assumptions were checked. The significance level alpha was set to 0.05.

## Results

### Subject characteristics

The neonatal characteristics and morbidities did not differ significantly between the NC+ and NC− groups; however, the NC+ group tended to be younger, smaller, and with more vancomycin, less prenatal steroid use, and more frequent IVH for all grades (Table 1). Children in the control group at visit were insignificantly older but significantly heavier, taller, and had larger head circumference, BSA, and LBM, but lower waist-to-height ratio. Between NC+ and NC−, only SDS for height and waist-to-height ratio were different at visit (Table 2). None of the kidney ultrasound investigations showed signs of persistent NC. One child from the control group was referred for follow-up ultrasound because of mild unilateral pelvic dilatation (excluded).

**Table 1:** Neonatal characteristics and morbidities for the three groups: Extremely preterm infants born < 28 weeks gestational age (EPT) with nephrocalcinosis (NC+) or without nephrocalcinosis (NC−) during the neonatal period and full-term controls. Values are presented as means and standard deviations (SD) or numbers or percent (*n* (%)) as specified

	EPT + NC *n* = 20	EPT − NC *n* = 21	Control *n* = 19	*p* value
Males, *n* (%)	9 (45%)	13 (62%)	10 (53%)	0.55/0.27
Gestational age, weeks	25.5 (1.2)*	25.9 (1.3)*	39.7 (1.6)	< 0.0001/0.22
Birth weight, g	755 (124)*	841 (202)*	3586 (477)	< 0.0001/0.10
Birth weight, SDS	− 0.93 (0.78)*	− 0.87 (1.22)*	0.19 (0.93)	0.0012/0.85
Birth length, cm	32.4 (1.8)*	33.6 (2.6)*	50.4 (1.9)	< 0.0001/0.08
Head circumference, cm	23.3 (1.3)*	24.0 (1.8)*	34.6 (1.4)	< 0.0001/0.12
Small for gestational age, *n* (%)	3 (15%)*	5 (24%)*	0 (0)	0.028/0.47
Apgar score at 5 min	6.7 (2.9)*	7.4 (2.1)*	10 (0.0)	0.0007/0.36
Apgar score at 10 min	8.4 (1.7)*	8.7 (1.9)*	10 (0.0)	0.022/0.59
BPD, *n* (%)	12/20 (60%)	10/21 (48%)	–	0.42
PDA (treated + untreated), *n* (%)	17/20 (85%)	17/21 (83%)	–	0.7
PDA, medically treated, *n* (%)	10/17 (59%)	13/17 (68%)	–	0.2
PDA, surgically treated, *n* (%)	9/17 (53%)	8/17 (47%)	–	0.7
ROP (stage III or >), *n* (%)	3/20 (15%)	2/21 (9.5%)	–	0.6
NEC (stage II or >), *n* (%)	11/20 (55%)	11/21 (54%)	–	0.8
NEC, surgically treated, *n* (%)	3/11 (27%)	4/11 (32%)	–	0.6
Sepsis, *n* (%)	11/20 (55%)	14/21 (61%)	–	0.4
IVH (I-II), *n* (%)	10/20 (50%)	6/21 (29%)	–	0.15
IVH (III-IV), *n* (%)	4/20 (20%)	1/21 (5%)	–	0.13
AKI (all stages), *n* (%)	8/20 (40%)	12/21 (57%)	–	0.3
Duration of aminoglycosides, days	17.1 (6.4)	16.2 (11.2)	–	0.8
Vancomycin treatment, *n* (%)	18/20 (90%)	15/21 (71%)	–	0.1
Duration of vancomycin, days	16 (9)	22 (11)	–	0.14
Furosemide treatment (iv/po), *n* (%)	18/20 (90%)	19/21 (91%)	–	0.95
Duration of all furosemide, days	38 (32)	49 (56)	–	0.49
NSAID for PDA closure, *n* (%)	10/20 (50%)	13/21 (62%)	–	
Antenatal steroids: full course, *n* (%) Incomplete course	11/20 (55%) 7/20 (35%)	17/21(81%) 2/21(10%)	–	0.11
Postnatal inhaled steroids, *n* (%)	13/20 (65%)	15/21 (71%)	–	0.65
Postnatal systemic steroids, *n* (%)	4/20 (20%)	3/21 (14%)	–	0.62
All postnatal steroids, days	3 (24)	43 (50)	–	0.44

Statistics were done with one-way ANOVA for comparison of all three groups/and with Pearson’s chi-square test for NC+ versus NC−. Asterisk is indicating *p* values < 0.05 and considered significant. *SDS* standard deviation score, *BPD* bronchopulmonary dysplasia, *PDA* persistent ductus arteriosus, *ROP* retinopathy of the premature, *NEC* necrotizing enterocolitis, *IVH* intraventricular hemorrhage, *AKI* acute kidney injury, *NSAID* nonsteroidal anti-inflammatory drugs

**Table 2:** Characteristics at follow-up visit for the three groups: Extremely preterm infants born < 28 weeks gestational age (EPT) with nephrocalcinosis (NC+) or without nephrocalcinosis (NC−) during the neonatal period and full-term controls. Values are presented as means and standard deviation (SD)

	EPT NC+ *n* = 20	EPT NC− *n* = 21	Control *n* = 19	*p* value ANOVA/NC+ vs NC−
Age at visit, years	7.8 (1.0)	7.4 (1.1)	8.1 (1.2)	0.1/0.2
Body weight, kg	22.5 (5.8)*	22.3 (5.4)*	26.7 (4.0)	0.02/0.9
Body weight SDS	− 1.26 (1.5)*	− 0.87 (1.3)*	− 0.02 (0.7)	0.01/0.4
Body height, cm	120 (7.6)*	121 (8.3)*	129 (8.6)	0.0005/0.5
Body height SDS	− 1.2 (1.2)*	− 0.5 (0.9)*^#^	0.2 (0.7)	0.0001/0.03
Head circumference, cm	51.1 (1.6)*	51.8 (2.2)*	53.4 (1.6)	0.0009/0.2
Waist circumference, cm	58.0 (7.4)	55.5 (5.5)	57.7 (3.3)	0.3/0.2
Waist-to-height ratio	0.48 (0.05)*	0.45 (0.03)^#^	0.44 (0.02)	0.005/0.04
Body mass index	15.5 (2.4)	14.9 (1.8)	15.7 (1.2)	0.4/0.4
Body mass index SDS	− 0.7 (1.4)	− 0.9 (1.6)	− 0.2 (0.8)	0.3/0.6
Body surface area	0.86 (0.13)*	0.86 (0.12)*	0.98 (0.11)	0.003/0.9
Lean body mass (*n* = 12/16/17)	15.8 (2.7)*	15.6 (3.0)*	19.6 (3.5)	0.001/0.8

*Significant difference between the three groups using one-way ANOVA (*p* values < 0.05). ^#^Significant differences (*p* values < 0.05) between NC+ and NC− born children using Pearson’s chi-square test

### Kidney volume

**Fig. 2:**
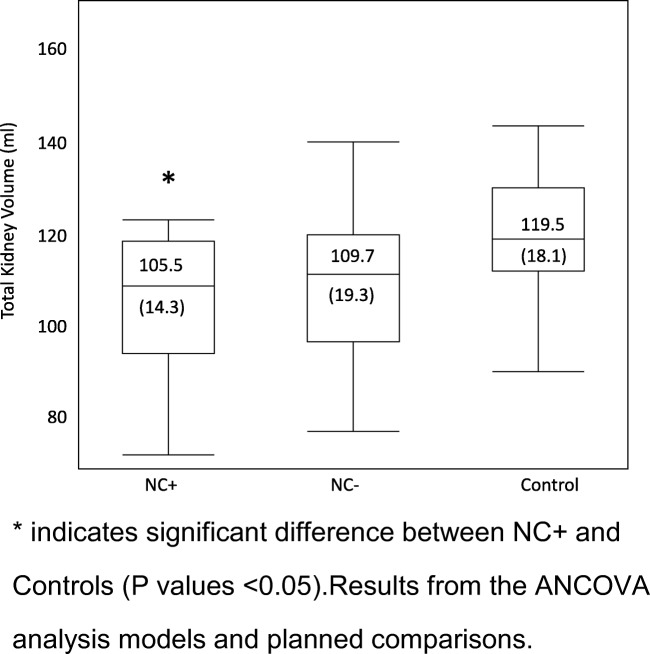
Total kidney volume presented as a ratio to body surface area (KV/BSA) for the three groups. Children born preterm screened positive for nephrocalcinosis (NC+), screened negative for nephrocalcinosis (NC−), and healthy term controls without nephrocalcinosis. *Significant difference between NC+ and controls (*p* values < 0.05). Results from the ANCOVA analysis models and planned comparisons.

**Fig. 3:**
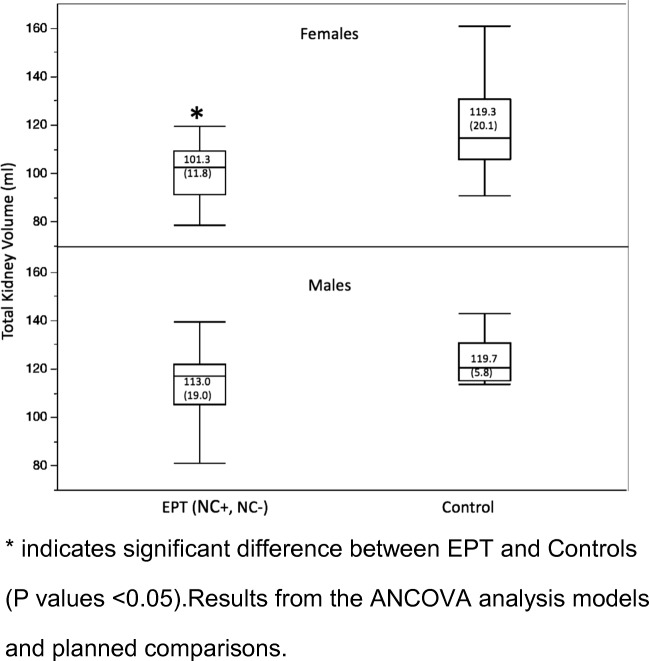
Total kidney volume presented as a ratio to body surface area (KV/BSA) for children born extremely preterm (EPT, both NC+ and NC−) and controls. *Significant difference between EPT and controls (*p* values < 0.05). Results from the ANCOVA analysis models and planned comparisons

Unadjusted total kidney volumes (KV) were significantly lower for both preterm groups in comparison with controls (NC+ = 90.1 ml, NC− = 93.8 ml, control = 118.4 ml, *p* = 0.0004, ANOVA, Fig. 2). After adjusting KV for BSA, the analysis was no longer significant between NC+ and controls (*p* = 0.056) (Table 3). The mixed effects model analysis where BSA, gender, and each kidney side were included showed a significantly lower right and left KV for girls in the NC+ group compared with girls in the control group (*p* = 0.016). The effects of the following factors on total KV by using stepwise linear regression were also tested: age at visit, AKI, PDA, NEC, BPD, sepsis, and treatment with furosemide (days) and antenatal steroids. None of the tested confounding factors seemed to have the potential to explain a difference in total KV between the two preterm groups. Total KV calculated as the ratio of KV and BSA (KV/BSA) [26] was significantly lower for the NC+ group of preterm-born children compared with controls (*p* = 0.016), but not reaching significance for the NC− group (*p* = 0.08) (Table 4, Fig. 2). Both preterm groups taken together had significantly lower kidney volume in comparison with controls (Table 4). There was no significant difference between NC+ and NC− groups. Regardless of the method of the statistical analysis, there were differences in kidney volume between boys and girls (Fig. 3) as well as laterality of the kidney shown in Tables 3 and 4. Total KV calculated as a ratio to BSA (KV/BSA) for the neonatal period was not significantly different between infants with or without NC (NC+: 131.4 ml (SD 21.1); NC− 109.9 ml (SD 29.7), *p* = 0.07 using non-parametric Kruskal-Wallis test). The KV/BSA ratio from the neonatal period compared with the measurements at school age showed that children who had suffered from NC (NC+) during the neonatal period had significantly lower KV/BSA ratios than those without NC (NC−) (NC+ 81.05, NC− 103.4; *p* = 0.0036). Among NC+ children, only 2 of 18 (11%) had a rise in KV/BSA ratio from neonatal to school age, while 9 of 18 (50%) in the NC− group showed a rise in KV/BSA ratio (*p* = 0.01).

**Table 3:** Statistical results for kidney volume adjusted for BSA comparing the different groups: extremely preterm infants born < 28 weeks gestational age with nephrocalcinosis (NC+) or without nephrocalcinosis during the neonatal period (NC−) and full-term controls

Kidney volume adjusted for BSA at visit	Estimated difference (CI 95%)	*p*
Total kidney volume		
NC+ vs NC−	− 2.97 (− 13.09 + 7.14)	0.55
NC+ vs control	− 11.12 (− 22.57 + 0.31)	0.056
NC− vs control	− 8.15 (− 19.4 + 3.06)	0.15
(NC+ NC−) vs control	− 9.64 (− 19.79 + 0.51)	0.062
Right kidney volume		
NC+ vs NC−	− 4.72 (− 10.31 0.86)	0.09
NC+ vs control	− 8.7 (− 14.9–2.5)	0.0068*
NC− vs control	− 3.97 (− 10.09 2.13)	0.19
(NC+ NC -) vs control	− 6.34 (− 11.83–0.85)	0.024*
Left kidney volume		
NC+ vs NC−	2.23 (− 5.25 9.72)	0.55
NC+ vs control	− 5.16 (− 13.47 3.1)	0.21
NC− vs control	− 7.4 (− 15.59 0.79)	0.07
(NC+ NC−) vs control	− 6.28 (− 13.63 1.07)	0.09
Total kidney volume girls		
NC+ vs NC−	7.66 (− 6.93 + 22.25)	0.28
NC+ vs control	− 14.76 (− 29.04–0.49)	0.04*
NC− vs control	− 22.42 (− 38.76–6.08)	0.009*
(NC+ NC−) vs control	− 18.59 (− 32.09–5.1)	0.009*
Total kidney volume boys		
NC+ vs NC−	− 6.83 (− 21.88 + 8.22)	0.36
NC+ vs control	− 6.7 (− 25.29 + 11.89)	0.46
NC− vs control	0.12 (− 15.63 + 15.94)	0.98
(NC+ NC−) vs control	− 3.28 (− 18–81 + 12.24)	0.66

Results from the ANCOVA analysis models and planned comparisons. *p values are regarded as significant (*p* values < 0.05)

**Table 4:** Results for kidney volumes using BSA-related KV (KV/BSA) for the different groups

	Estimated difference (CI 95%)	*p*
Total Kidney volume (KV/BSA)		
NC+ vs NC−	− 4.21 (− 15.29 + 6.86)	0.44
NC+ vs control	− 14.02 (− 25.37–2.66)	0.016*
NC− vs control	− 9.8 (− 20.88 + 1.27)	0.08
(NC+ NC−) vs control	− 11.8 (− 21.51–2.09)	0.018*
Right kidney volume (KV/BSA)		
NC+ vs NC−	− 5.04 (− 11.0 + 0.93)	0.09
NC+ vs control	− 9.21 (− 15.33–3.08)	0.003*
NC− vs control	− 4.16 (− 10.22 + 1.89)	0.17
(NC+ NC−) vs control	− 6.62 (− 12.01–1.23)	0.016*
Left kidney volume (KV/BSA)		
NC+ vs NC−	− 2.4 (− 10.9 + 5.96)	0.55
NC+ vs control	− 6.99 (− 15.64 + 1.64)	0.11
NC− vs control	− 9.47 (− 18.01–0.92)	0.03*
(NC+ NC−) vs control	− 8.26 (− 15.7–0.82)	0.03*

Results from the ANCOVA analysis models and planned comparisons. *p values are regarded as significant (p values < 0.05)

### Kidney function

All groups had normal estimated glomerular filtration rate (eGFR) calculated by the cystatin C–based CAPA formula but showed lower values for the NC− group compared with controls as well as for both preterm groups taken together (NC+ 120, NC− 113, control 126.3 ml/min/1.73m^2^, *p* = 0.012, 0.035 respectively). The difference between the NC+ and NC− groups was not significant (*p* = 0.11).

There was no difference between the groups when using the simplified Schwartz formula (eGFRcreatinine with *k* = 36) (NC+ 114.4, NC− 112.5, control 104.6 ml/min/1.73m^2^, *p* = 0.3). Although entirely normal, plasma creatinine was significantly higher in controls compared with both groups of preterm-born children (NC+ 38.5 (5.9); NC− 40.1 (7.9); control 46.2 μmol/l (8.6), *p* = 0.011). Urinary proteins and electrolytes were not significantly different between the three groups (data on request).

### Blood pressure

Office blood pressure and systolic and diastolic standard deviation score (SDS) were not different between the three groups (mean (SD); NC+, 0.18 (0.94); NC−, − 0.07 (0.72); control, − 0.005 (0.72), *p* = 0.58). Four of the preterm-born children had high office blood pressure measurements: 3 had both systolic and diastolic, and 1 had isolated diastolic blood pressure above the 90th percentile. One child in the control group had isolated high diastolic blood pressure measurements above the 90th percentile.

ABPM readings were successful in 34 of the 41 EPT-born children. Neurodevelopmental disorders of autism spectrum disorders and/or ADHD were present in 16 children born EPT (12/20 NC+ (60%); 4 of 21 NC− (19%)). In seven of these children, ABPM measurements were not possible due to compliance problems or had to be discarded because of low quality. However, none of the investigated children with ADHD or autism spectrum diseases was on medical treatment at the time of investigation. The results of ABPM in the 34 performed were in the normal range for all preterm-born children. ABPM could verify the high office blood pressure measurements in only one of the five patients mentioned above. The majority of the children had systolic values below or at the 50th percentile (Table 5). There was no difference between NC+ and NC− with regard to the distribution among the percentiles. Seventeen of 34 preterm-born children (50%) (NC+ 9; NC− 8) had a day-to-night decline (night dipping) of < 10% (Table 5).

**Table 5:** Results for ABPM (presented as numbers for percentiles) for the three groups

	NC+ (14)	NC− (20)	*p*
24-h ABPM systolic percentiles			
< 50th	9	14	0.7
50–75th	5	3	0.2
75–90th	0	3	0.1
> 90th	0	0	
24-h ABPM diastolic percentiles			
< 50th	13	17	0.5
50–75th	1	2	0.7
75–90th	0	1	0.4
> 90th	0	0	
Day-to-night decrease < 10%			
Patients, *n* (%)	9 (64.3%)	8 (40%)	0.16

Differences between NC+ and NC− groups analyzed with Pearson’s chi-square test. The normal percentiles are taken from reference [22]

## Discussion

After adjusting KV for BSA, we can show that extremely preterm-born children already at early school age have significantly smaller kidneys than their peers born at term. We cannot prove that NC has a clear effect on those findings, but we can show that mainly preterm-born children exposed to NC are the ones with smaller kidneys in comparison with controls. We were also able to show that the evolution of the KV/BSA ratio from the neonatal period to school age seems to be negatively affected for those who suffered from NC during their neonatal period. It remains speculative whether NC during the neonatal period has an adverse effect on kidney growth. We fully appreciate that the number of patients included does not allow any causative relation. However, we regard this observation as important and interesting.

Kidney function and office blood pressure and ABPM were normal and not different between the groups. However circadian blood pressure regulation seemed to be significantly altered in both groups of preterm-born children.

With regard to NC, Giapros et al. followed children born under 32 weeks GA to the age of 24 months and demonstrated that those born preterm who had developed NC had shorter right-sided kidney length [9]. Kist-van Holthe et al. found smaller kidneys in preterm-born children at the age of 7.5 years but no effect of NC on kidney volume.

The association between prematurity and smaller kidneys in infants and toddlers up to childhood has been shown in a number of follow-up studies [27, 28]. Other long-term follow-up studies, including our own previous research, showed somewhat conflicting results [29]. Kwinta and later Stazec et al. could confirm reduced kidney volume measured by ultrasound in very low birth weight (VLBW) children at the age of 7 and 11 years [30, 31]. A study on renal biopsy material in 31 children with focal segmental glomerulosclerosis (FSGS) or minimal change nephrotic syndrome (MCNS) at a mean age of 11 years, where 8 children also were born very preterm (mean: 25.4 GA), showed significantly lower glomerular density and greater glomerular volume in the children born preterm compared with those with normal birth weight [32]. The vicious circle of fewer nephrons in children born with low birth weight introduced by Brenner et al. might be particularly important in patients where there already exists severe renal pathology [33].

Our findings also show that school-aged girls born EPT have smaller kidneys than controls—a finding not present for boys. Keijzer-Veen et al. also found preterm born females to be more affected than males at young adulthood [34].

Kidney function was, by definition, normal among all three groups. Cystatin C–based eGFR, which has been advocated to be more appropriate for the age group investigated here, was lower in the group of children born prematurely in comparison with the control group (*p* = 0.036) [35]. Using the modified Schwartz formula to establish eGFR, four children in the preterm groups and one child from the control group had eGFR below 85 ml/min/1.73m^2^, which can be defined as mild renal insufficiency [36]. Serum creatinine levels were higher in the control group which might be explained by the higher muscle mass in that group. We could not detect any difference between preterm-born children with or without NC in regard to eGFR. This is in slight contrast to the findings in the study by Kist-van Holthe et al. who showed a higher number of children in their NC+ group with mild chronic renal insufficiency, but these results were not significantly different to the children born preterm without NC [17]. Giapros et al. could not detect an effect on GFR in his follow-up study focusing on children born preterm up to 24 months of age [9]. Follow-up studies of the same cohort at the age of 15, 20, and 30 years of age will be informative and necessary to answer the abovementioned statements.

Early elevated blood pressure in children born very preterm, and even hypertension, has been reported in previous studies [37, 38]. It is debatable whether office blood pressure measurements are capable of reflecting true elevation of blood pressure or if there is a risk for overestimation. When we measured 24-h blood pressure in the preterm-born children, only one of the 5 children with office blood pressure above the 90th percentile had also elevated blood pressure in the ABPM.

The results of the ambulatory blood pressure measurements were within the normal limits for systolic and diastolic age and height related percentiles (< 90th) [22]. However, we were very surprised by the high number of “non-dippers” among the children born premature. In 17 out of 34 (50%) children with reliable 24-h readings, the blood pressure difference between day and night time was less than what is generally regarded as normal (10% difference) [39]. The relevance of “non-dippers” has been well described for adults and has been strongly associated with worse cardiovascular outcome and can be interpreted as a marker preceding the development of hypertension and microvascular complications [40, 41]. There is very limited data available for children on night-dipping, but a few studies confirm that the 10% rule should be valid for children in the age group we investigated [22]. Night dipping seems to be related to age. Varda et al. observed in infants and toddlers from 2 to 30 months old a less pronounced night dip of only 5.4% on average [42]. A recent study observed a close relationship between non-dippers and BMI with children with primary hypertension and overweight or obesity showing a lack of decline of nocturnal blood pressure values [43]. Unfortunately, that study lacks information on GA or birth weight. We are unable to explain the “non-dipping” phenomena with overweight or obesity in our study as only 4 out of the 17 non-dipper children had a BMI at or over the 90th percentile.

Among the scarce evidence available for preterm-born children, a study in 41 preterm-born children (26–36 weeks GA) examined at the age of 7 years found in comparison with 27 healthy controls insufficient night dipping in 73% of the preterm group compared with 41% in the control group [44]. It is rather unclear why the prevalence of non-dipping is so high among healthy control children in this study. Another recent and slightly larger study investigated 78 preterm-born children (27 weeks mean GA) and compared them with 38 healthy term control children at the mean age of 6.7 years, but found only 16.7% versus 5.2% non-dippers [30]. Hovi et al. investigated 118 young adults (18 to 27 years of age) born with VLBW and could identify 31.2% “non-dippers” among those, along with the main findings of increased 24-h systolic blood pressure of 2.4 mmHg in young adults born with VLBW [45].

The variation of blood pressure over 24 h is regulated by the autonomic nervous system (ANS) via the hypothalamo-pituitary-adrenal axis [46]. It has recently been shown by us and others that the ANS might be altered in children born very premature [47–49]. However, the normal or even relatively low blood pressure during daytime in some of our preterm-born school children might reflect physical inactivity during the day and not allowing further dipping during nighttime. Although we are convinced that these data are of importance, they have to be interpreted with caution and further follow-up on this cohort is needed.

A general weakness of this study is the size, as the numbers in all three groups are limited. We regard it as a strength that we have analyzed KV with different methods and have thoroughly tested adjusting for different variables. Our investigation and analysis allowed us to make clear differentiations for gender as well as for right and left kidney sides. It was also beneficial that all study subjects were recruited from the same hospital which minimized confounding effects by different practices and that all histories and ultrasound examinations were performed by the same investigator, as well as all anthropometric measurements being taken by the same research nurse. ABPM were only taken in the EPT group and thus correlated to population blood pressure references which is another weakness.

## Conclusion

In this study, we showed that children at the age of 6–10 years born EPT have significantly smaller kidney volume and lower cystatin C–based GFR, but within normal limits. Our results do not entirely support our hypothesis that NC which developed during the neonatal period has a significant impact on reduced kidney volume at school age. However, the NC+ group of EPT-born children had significantly lower renal growth. Kidney function at school age has not been affected by NC. The high number of non-dippers in preterm-born children at school age is a new observation and has potential implications for the development of hypertension. However, the clinical significance of this finding needs to be studied further. It is important to know when early morphological changes such as the reduced nephron endowment lead to clinical findings in children born prematurely. As more EPT infants are surviving, research describing the consequences is essential in order to be prepared for adequate support and to organize early preventive efforts for this high-risk group.

## Abbreviations

ABPM: Ambulatory blood pressure measurements
BMI: Body mass index
BSA: Body surface area
EPT: Preterm
KV: Kidney volume
NC: Nephrocalcinosis
NC+: Nephrocalcinosis positive
NC−: Nephrocalcinosis negative
